# Enabling pregnant women and their physicians to make informed medication decisions using artificial intelligence

**DOI:** 10.1007/s10928-020-09685-1

**Published:** 2020-04-11

**Authors:** Lena Davidson, Mary Regina Boland

**Affiliations:** 1 Department of Biostatistics, Epidemiology and Informatics, Perelman School of Medicine, University of Pennsylvania, 423 Guardian Drive, 421 Blockley Hall, Philadelphia, PA 19104 USA; 2 Institute for Biomedical Informatics, University of Pennsylvania, Philadelphia, USA; 3 Center for Excellence in Environmental Toxicology, University of Pennsylvania, Philadelphia, USA; 4 Department of Biomedical and Health Informatics, Children’s Hospital of Philadelphia, Philadelphia, USA

**Keywords:** Literature review, Pregnancy, Artificial intelligence, Machine learning, Decision support systems

## Abstract

The role of artificial intelligence (AI) in healthcare for pregnant women. To assess the role of AI in women’s health, discover gaps, and discuss the future of AI in maternal health. A systematic review of English articles using EMBASE, PubMed, and SCOPUS. Search terms included pregnancy and AI. Research articles and book chapters were included, while conference papers, editorials and notes were excluded from the review. Included papers focused on pregnancy and AI methods, and pertained to pharmacologic interventions. We identified 376 distinct studies from our queries. A final set of 31 papers were included for the review. Included papers represented a variety of pregnancy concerns and multidisciplinary applications of AI. Few studies relate to pregnancy, AI, and pharmacologics and therefore, we review carefully those studies. External validation of models and techniques described in the studies is limited, impeding on generalizability of the studies. Our review describes how AI has been applied to address maternal health, throughout the pregnancy process: preconception, prenatal, perinatal, and postnatal health concerns. However, there is a lack of research applying AI methods to understand how *pharmacologic* treatments affect pregnancy. We identify three areas where AI methods could be used to improve our understanding of pharmacological effects of pregnancy, including: (a) obtaining sound and reliable data from clinical records (15 studies), (b) designing optimized animal experiments to validate specific hypotheses (1 study) to (c) implementing decision support systems that inform decision-making (11 studies). The largest literature gap that we identified is with regards to using AI methods to optimize translational studies between animals and humans for pregnancy-related drug exposures.

**Electronic supplementary material:**

The online version of this article (10.1007/s10928-020-09685-1) contains supplementary material, which is available to authorized users.

## Introduction

### Artificial intelligence and informing healthcare decision making

The field of artificial intelligence (AI) involves the study of ‘agents’ that receive information from their environment and perform actions in response to that environment [1]. These ‘agents’ are sometimes referred to as ‘intelligent agents’ [1]. In general, AI is used to refer to the method by which computer systems can perform tasks that would typically require a human. This includes tasks such as translating documents into different languages, automatically identifying a person from an image (visual perception) or decision-making. In this review, we will focus our discussion on both clinical and patient decision-making, as these are two areas where AI has the potential to impact decision making with respect to pharmacological or drug choices during pregnancy.

Initially AI methods that were used in healthcare focused around rule-based decision-making. AI tools that utilized rule based decision-making fit naturally within the clinical environment because they can effectively mirror the clinicians’ own decision-making process. One of the first rule-based decision-making algorithms was MYCIN. MYCIN was developed in 1974 to predict the appropriate therapy for different bacterial infections [2]. It was designed as an ‘expert system’ that would guide clinicians to appropriate decision making, using a series of if–then statements. These ‘expert’ rule-based systems would be first applied in the field of women’s health some 20 years later in 1994 with the development of a rule-based pre-term birth risk predictor. This rule-based system predicted a woman’s risk of preterm birth using diagnostic codes during the pregnancy and utilized the ‘state-of-the-art’ in AI at that time [3]. These types of programs can only achieve improved performance through restructuring. Machine learning (ML) is an application of AI that enables learning without being explicitly programmed. A popular method of ML, an artificial neural network (ANN) is designed to resemble how biological neural systems process data.

### AI and machine learning defined

AI is the broad science of mimicking human abilities. Machine learning is a subset of AI, in the field of computer science. ML often uses statistical techniques to allow for the computer to “learn”, or progressively improve performance on a given task, without being explicitly programmed. ML refers to a number of methods and algorithms, and different learning types: supervised, semi-supervised, unsupervised, reinforcement, evolutionary, and deep learning [4, 5]. In supervised learning, every input pattern is trained to an associated output pattern and error in computed and desired outputs can be used in improve performance. Common supervised learning algorithms include regression and classification algorithms, such as the following: simple linear regression, polynomial regression, LASSO regression, k-Nearest Neighbors, Support Vector Machines (SVM), Naïve Bayes (NB), Decisions Trees (DT), and Random Forests (RF). In unsupervised learning, the network trains without knowledge of the desired output. Common unsupervised learning methods include clustering algorithms and dimension reduction algorithms, such as: k-means clustering, principal component analysis (PCA), and independent component analysis (ICA). In reinforcement learning, agents are not presented and the desired output is learned from the actions that are the best through trial and error [6]. ML models learn from a given dataset, with instances and features; an instance is an individual or example in the data. Each instance has a number of features, or attributes, describing an aspect of that instance. See Table 1 for an overview of ML model abbreviations.

**Table 1:** Artificial intelligence abbreviations

Abbreviation	Description
ANN	Artificial neural network
CART	Classification and regression tree
CDSS	Clinical decision support system
DL	Deep learning
DT	Decision tree
EM	Expectation-maximation
k-NN	k nearest neighbor
LDA	Linear discriminant analysis
LR	Logistic regression
MLP	Multi-layer perceptron
NB	Naïve bayes
RF	Random forest
RBF	Radial basis function
SVM	Support vector machines

### Pros and cons of ML models

Important care needs to be taken when considering different ML techniques for a classification problem. However, this decision is situational and dependent on the dimensionality, size, and other qualities of the dataset. ML methods are not designed to demonstrate causality, and at best can provide likely candidates for causality. No single model performs optimally across all problems and this phenomena is called the No Free Lunch theorem. For this reason, it is common to compare more than one modeling approach, compare models with different parameters, or develop an ensemble approach. For the sake of this review, we will not delve deeply into advantages and disadvantages among AI methods. A recent perspective article provides an overview of the barriers to deployment and translational impact of ML methods for health care [7]. The operation and fitting of ML methods [8], the ethics of AI in medicine [9], as well as unintended consequences [10, 11] are comprehensively discussed elsewhere.

### Artificial intelligence, maternal and fetal health, and pharmacological intervention

This systematic literature review focuses on understanding the current research on AI methods as applied during pregnancy with a focus on optimizing drug/pharmacological therapies among pregnant women. We also identify gaps in the field where AI can be used more in the future.

## Methods

We used PRISMA guidelines when conducting our literature review [12].

### Systematic review of literature

Our systematic literature review focuses on the role of AI in maternal healthcare. Our first step was to search for relevant literature articles pertaining to AI and pregnancy. We searched 3 databases, EMBASE, PubMed and SCOPUS. PubMed contains research funded by the National Institutes of Health from the United States of America. EMBASE is a biomedical and pharmacological bibliographic database of published literature with a primary focus on pharmacovigilance. SCOPUS is Elsevier's abstract and citation database covering articles from 34,346 peer-reviewed journals. We used site licenses from the University of Pennsylvania libraries to search SCOPUS and EMBASE. We used the following search query to identify papers within the described scope:

“artificial intelligence” and “pregnancy”.

The PubMed interface automatically maps search words to their respective Medical Subject Headings terms using Automatic Term Mapping [13]. After retrieving results from each database, we removed duplicate studies using exact PubMed ID match. We then manually reviewed articles and compared title, author list and publication date to further identify duplicate publications in the case where PubMed IDs were absent. In some cases, a paper was listed in EMBASE and SCOPUS, but not in PubMed and therefore no ID was available for comparison purposes. We filtered the results further by excluding non-English studies, conference papers, editorials, and notes.

**Fig. 1:**
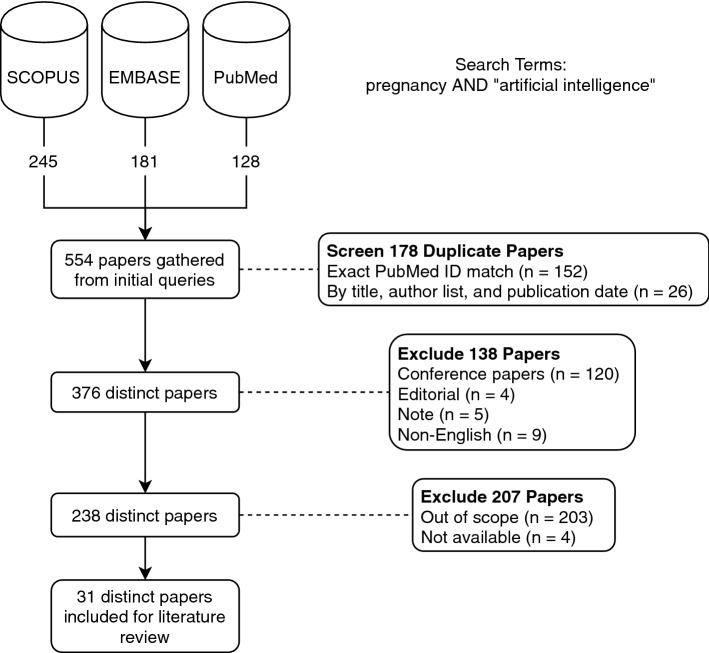
Prisma review

L.D. manually reviewed all of these articles and categorized them by focus and domain. The eligibility criteria considered research within an unrestricted range of years, encompasses AI and maternal health, and pertains to pharmacologic interventions. No unpublished relevant papers were retained. See flow diagram Fig. 1 for an overview of our review methodology.

## Results

### Systematic review of literature

We searched EMBASE, PubMed and SCOPUS for articles on pregnancy and AI. Our query from December 5, 2019 found 245 from SCOPUS, 181 from EMBASE, and 128 from PubMed. We removed duplicate studies retrieved from across databases using exact PubMed ID match. We started with the set of 245 papers from SCOPUS and found an additional 120 from EMBASE, an additional 33 papers in PubMed not found in SCOPUS, and 4 papers found in references from retrieved papers. We manually reviewed these to further identify duplicate papers. We identified duplicates by title, publication date and author list information. Next, we excluded non-English studies: 9 were non-English with 6 in German, 1 in Polish, 1 in Chinese, and 1 in Portuguese. We also excluded 119 conference papers, 4 editorials, and 5 notes, resulting in a set of 238 research papers.

Subsequently, articles were assessed for eligibility. The 238 articles were manually reviewed to determine if they met selection criteria. Namely, the articles retained for further review: (1) focused on AI; (2) related to pregnancy; and (3) included or pertained to pharmacologic treatment in the study. These inclusion criteria resulted in 31 relevant papers, 4 of which were reviews of AI applications in pregnancy care.

**Fig. 2:**
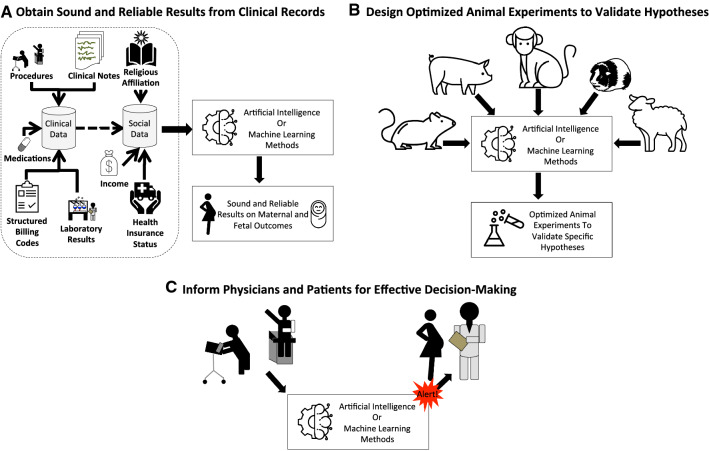
Artificial intelligence and machine learning can enable better informed decisions for pregnant women in multiple ways. **a** illustrates how artificial intelligence and machine learning couple be utilized to process multiple data modalities present in clinical data to derive sound and reliable results pertaining to maternal and fetal outcomes. **b** illustrates how artificial intelligence or machine learning methods could be used to design optimal animal models for experiments that validate retrospective findings obtained from clinical records or other sources. **c** illustrates how artificial intelligence or machine learning methods could be used to alert physicians and their patients at the appropriate time pertaining to specific details related to pregnant or nursing women

The final set includes articles issued from 1990 to 2019, encompassing almost 3 decades of research. Papers are shown summarized by overarching category (Table 2), either A, B, or C, which are detail in Fig. 2.

**Table 2:** Studies by identified category and overall topic

Category	Topic	References	# of papers
A: Analysis with clinical data	ART	[14–20]	7
	Biomarkers	[21]	1
	Imaging	[22]	1
	Prediction	[23–28]	6
	Total		15
B: Translating results from animals models humans	Translational	[29]	1
	Total		1
C: Clinical decision support/alerting	Diagnosis	[30–32]	3
	Disease management	[33–36]	4
	Pregnancy outcome	[37]	1
	Expert system	[38–40]	3
	Total		11
Other	Review	[41–44]	4
Total count			31

### Identified applications of AI in pregnancy care and pharmacologics

Selected papers include research from a variety of disciplines: computer science, engineering, informatics, teratology, pharmacy science, health information systems, and general medicine and biology. Look to Table S1 for an overview of the variety of journals from the included papers. The research encompasses AI applications at several stages of pregnancy, including preconception and assisted reproductive technologies; prenatal care and pregnancy induced disease; birth and delivery; and postpartum disease. Assisted reproductive technologies (ART) and prediction of pregnancy-associated complications are more represented in our review, only one paper using biomarkers and one paper applying AI to imaging during pregnancy. More research pertaining to AI imaging research was found in our query, however the research involved fetal brain analysis, cervical properties, and embryo selection. Therefore, those studies were excluded, and a single study using AI methods to determine pharmacological intervention placement was included. Similarly, one paper was found focusing on applying AI methods to improve translational research. Several clinical decision support system (CDSS) studies were found, with more focus on diagnosis, disease management, and expert systems for clinical use. One paper focused on prediction of birth outcomes, while there were other papers focused on this from our query search, this was the only paper that incorporated pharmacological intervention data into the methodology.

## Discussion

### Better ‘actionable’ science needed for pregnant women

Twenty-five years have passed since the first AI tool was developed for a woman’s health issue (preterm birth) and a full 45 years since the first health-based ‘expert’ AI system was developed. However, much remains to be done in the realm of harnessing AI methods to improve healthcare, especially women’s health. Recently, two articles in the *New England Journal of Medicine* highlighted the important need for novel methods to investigate pharmacological effects among pregnant women [45] and also nursing or lactating women [46]. Both Eke et al. and Mitchell et al. note that 70–80% of pregnant women receive a pharmacologic during their first trimester of pregnancy (the most critical period with regards to congenital anomalies and adverse *fetal* outcomes) and 90% of pregnant women take a pharmacologic at any point during their pregnancy [45, 47]. Moreover, prescription medication use increases with maternal age and education [47]. However, the majority of these medications are prescribed and taken without any randomized controlled trials that include pregnant women and this limits the ability for clinicians to understand the potential adverse health consequences both for the mother and the fetus [45]. A tremendous need exists to understand the effects of pharmacologics not only on the developing fetuses in terms of anomalies and other adverse fetal consequences, but also with regards to the mother. Adverse maternal consequences are possible and include excessive bleeding and other perinatal and postpartum complications.

Pregnant and postpartum women have been systematically excluded from research due to their vulnerable status [48]. Pregnant women may be viewed as scientifically complex, and there are practical and ethical issues surrounding the inclusion of pregnant and lactating women in clinical trials. The Task Force on Research Specific to Pregnant Women and Lactating Women (PRGLAC) wrote a report to the secretary of health and human services and Congress in September 2018, outlining strategies for identifying and addressing gaps in knowledge and research regarding drug use of pregnant and lactating women [49]. Because of this gap in scientific knowledge on the effects of treatments for the health needs of pregnant and lactating women, fair inclusion implies that a boost of research in this population is warranted [50]. While efforts in trial design are discussed [45], application of AI in this domain remains lacking.

The state of maternal healthcare in the USA is currently at a cusp, maternal mortality is increasing despite decreases observed worldwide [51]. In addition, the pharmacological effect of drugs taking during pregnancy still remains largely unknown and underexplored. Clearly better science is needed—and this must go beyond the inclusion or exclusion of pregnant women in clinical trials as suggested by Eke et al. [45]. Rather better AI methods are needed that appropriately harness both the existing data—in terms of Electronic Health Records (EHRs) and also toxicological and chemical data from the pharmacologics themselves.

### Recent uses of AI to understand maternal and fetal health outcomes from pharmacologics

A major challenge for methods that seek to understand the *fetal* and maternal consequences of pharmacologics taken during pregnancy is that few randomized controlled trial data exist in humans. The focus tends to be on at-risk populations, such as opioid substitution therapy [52], HIV prevention therapy [53], and preterm birth [54]. Concerning the general pregnant population, there is a gap of research on medication use during pregnancy. Researchers must utilize data taken during routine clinical care, and therefore studies are often retrospective outcomes studies. In addition, while data are available in animal models there are major gaps in translating this information to the human context [55]. Therefore, data exists, but these data must be repurposed to answer important clinical questions. This is where AI methods can be especially powerful.

#### Animal models and pregnancy

Cox et al. conducted a proteomics study to breakdown preeclampsia (PE) patients into three distinct subgroups, based upon distinctive molecular differences [29]. This was achieved through translating enriched trophoblasts from mice to human PE placenta data. BayesNet ML was used to compensate for the contamination of subcellular compartments. The study suggests that maternal molecular pathologies from placentas could distinguish the three identified human subgroups of PE. Understanding the diversity in PE etiology should prove valuable for individualizing diagnostic and treatment regimes.

#### Assisted reproductive technologies

Research in ART is represented well due to the fact that pharmacological intervention is necessary for several common ART practices. AI methods have been applied to inform and advise physicians [14], to predict pregnancy success [15–18], to provide optimized treatment [19], and to understand miscarriage risk [20].

Navigating infertility and ART treatment often takes several treatments and can be cost-prohibitive, and therefore there is significant focus on the prediction of pregnancy success and applications to improve treatment. Kaufmann et al. applied neural networks to predict success for individual couples about to undergo in-vitro fertilization (IVF) treatment [15]. Neural networks were created using 4 variables: maternal age, number of eggs retrieved, number of embryos transferred, and embryo freeze status (i.e., fresh or frozen). The highest predictive success of the 8 neural networks was 60%, which may be due to the fact that the input information was not sufficient—there is likely an absence of important predictor variables from the data set. Jurisica et al. developed a case-based reasoning system that relies on context-based relevance assessment to assist in knowledge visualization, interactive data exploration and discovery in IVF [14]. This CDSS acts as an advisor to the physician and can help inform the treatment to improve success rate; using 39 attributes, the CDSS suggests the amount of hormonal stimulation for treatment and suggested day for triggering the ovulation.

Gianaroli et al. conducted a retrospective study and proposed a Bayesian network (BN) model to predict occurrence of a pregnancy, and implantation status (i.e., no implantation, single implantation, and twin implantation) [16]. Variables considered and divided into categories for the model include the following: maternal age, previous IVF, intracytoplasmic sperm injection (ICSI) cycles, grade of each embryo, insemination technique, maternal/uterine receptivity, embryo viability, and pregnancy. Maternal receptivity and embryo viability were only partially observed, and therefore the authors used the Expectation–Maximization (EM) algorithm to estimate parameters. The model predicted the occurrence of pregnancy with an area under the curve (AUC) of 0.72. However, the model requires validation from a prospective study and is possibly a simple model for the complexity of implantation, lacking inclusion of more relevant features. Hassan et al. propose a method to predict IVF pregnancy using a hill climbing feature selection algorithm coupled with automated classification using a variety of supervised machine learning classifiers [17]. Important attributes of the 25 features were chosen by a majority of the classifiers, including maternal age, indication of infertility factor, antral follicle counts, and number of mature eggs.

Güvenir et al. proposed a technique for estimating the success of an IVF treatment using a ranking-based algorithm [18]. The dataset contained 64 features, 52 of which were female features and the other 12 related to the male. While this method included features of baseline luteinizing hormone, baseline follicle-stimulating hormone, and baseline estradiol, the type of treatment is not used as a feature to estimate success. Their methods are more tailored to screening potential IVF clients to determine success from clinical features. Siristatidis et al. proposed a web-based system to assist clinicians to provide personalized treatment for subfertile couples and improve ART outcome [19]. The system relies upon an ANN model and a database system that combines several databases across the health information system, including an IVF database. An example of a set of parameters for the ANN model includes cycle characteristics (24 parameters), couple’s evaluation (12 parameters), female evaluation (30 parameters), and male evaluation (12 parameters). Notably, albumin, gonadotrophin, and metformin use are listed as parameters, along with cortisolone co-use and stimulation protocol. The proposed system would assist at several points of care during IVF treatment and inform the model to enhance its performance with each record.

Mora-Sánchez et al. propose a methodology to analyze Human Leukocyte Antigen haplotypes from couples with recurrent miscarriage and couples with histories of successful pregnancies [20]. The SVM classifier with a linear kernel was used to predict the recurrent miscarriage and healthy pregnancy classes. An implication of this research is that accurately assessing the risk of recurrent miscarriage associated with a given pair of gametes could improve gamete donor selection and therefore increase pregnancy success rates.

A reoccurring theme from these ART studies is that predicting pregnancy success is complex, and models lack sufficient features for the most accurate prediction. While some models consider pharmacologic interventions as a feature for predicting pregnancy viability, it is often not determined to be an important attribute. A number of ovulation induction treatments are commonly used in infertility treatments: estrogen antagonists, insulin sensitizing agents, gonadotrophins, and GnRH analogs [56]. Further ART research applying machine learning methods could include the type of ovulation induction treatment as a feature.

#### Developmental toxicology

Jelovsek et al. created a methodology to elicit a set of rules for developmental toxicologic hazard identification from a group of experts, for use in AI application [38]. As a result of the interviewing process, the authors gathered a set of rules. Then, experts reviewed the cumulative rule set and determined whether each rule is a confidence rule or an important rule; while the expert may have confidence that the rule is valid, it may be of little importance. There was significant disagreement between experts and the rule classification required further clarification to the experts, showing that classification boundaries were unclear. Overall, the authors determined six variables that contribute to an expert’s decision as to whether or not a compound or agent is a developmental toxicologic hazard: (1) human studies results, (2) animal studies results, (3) whether an active compound is present in the human, (4) physical structure similarity to a known human developmental toxicant, (5) mechanism of action similarity to a known toxicant, and (6) whether the compound is a known mutagen or direct cytotoxic agent. There is a need to elicit a set of rules that cover pharmacologic principles, including aspects such as dose amount, absorption, route of exposure, mechanism of action, timing of exposure, and drug/disease interactions.

#### Chronic disease management and pregnancy

Prevalence of maternal chronic disease has been increasing in the United States. The number of women presenting at hospitalized delivery with 1 or more chronic conditions rose from 66.9 to 91.8 per 1000 delivery hospitalizations between 2005–2006 and 2013–2014 [57]. Chronic hypertension, chronic respiratory disease, substance-use disorders, and pre-existing diabetes are disorders with the greatest increase of prevalence over time [57]. One paper was found relating to chronic disease and pregnancy outcome. Systemic lupus erythematosus (SLE) is a chronic autoimmune disease with unknown etiology, and different clinical manifestations, laboratory signs and prognosis. Pregnancy among SLE-affected women is highly associated with poor obstetric outcomes, namely fetal loss from spontaneous abortion or intrauterine death [58]. Paydar et al. developed a CDSS to predict pregnancy outcomes among SLE-affected pregnant women, namely spontaneous abortion or live birth [37]. Two ANNs were trained based on features selected by a binary logistic regression (LR) model: a multi-layer perceptron (MLP) model and radial basis function (RBF) model. Significant features selected included a variety of drug intervention (hydroxychloroquine, azathioprine, aspirin) and laboratory test features (proteinuria, haematuria, antibody levels) before and during pregnancy. After tenfold cross-validation, the MLP network was found to be the most accurate (91%) for prediction of spontaneous abortion or live birth of SLE-affected pregnancy.

Chronic illness can have an effect on fertility, pregnancy outcomes, maternal outcomes, and fetal outcomes. Ideally, preconception care should address the potential poor outcomes by screening and providing research informed care and pregnancy-specific management of chronic disease.

#### Pregnancy-induced disease

Pregnancy causes the body to go through significant changes, and unfortunately there are several maternal complications that can occur during this time. Our query found papers focused on gestational diabetes mellitus (GDM) [23, 24, 30, 33–36, 41], gestational hypertension disorders [21, 25, 26], and bacteriuria [31]. Studies aim to predict and classify disease in early pregnancy, improve screenings, and provide clinical decision support for disease management. GDM prevalence is as high as 9.2% in the United States [59]. Maternal GDM has been associated with adverse outcomes for offspring: impaired glucose intolerance [60, 61], macrosomia at birth [62], risk factor for long-term neuropsychiatric morbidity [63], and early onset cardiovascular disease (CVD) [64]. GDM has important characteristics that differentiate it from type 1 or type 2 diabetes mellitus (T2D): (1) patients have endogenous insulin secretion (inadequate), (2) a patient’s metabolic state changes continuously and requires frequent treatment adjustments, and (3) the short duration of the illness may impact patient education and knowledge in insulin self-management [33].

Hypertensive disorders of pregnancy affect approximately 10% of all pregnant women worldwide [65]. Gestational hypertension, or pregnancy-induced hypertension (PIH), is high blood pressure during pregnancy, without presence of proteinuria. In some cases, PIH can develop into preeclampsia (PE)—a pregnancy-related vascular disorder, affecting 2–8% of all pregnancies and is the leading direct cause of maternal mortality worldwide, after obstetrical hemorrhage [66, 67]. Thought to be a severe form of PE [68], Hemolysis, Elevated Liver enzymes, and Low Platelet (HELLP) syndrome worsens maternal and perinatal prognosis. HELLP syndrome occurs in about 0.2–0.8% of pregnancies [69]. This complication is associated with increased maternal risks: pulmonary edema, cardiac failure, hemorrhage, renal failure, liver hematoma, failure or rupture, and death. Like PE, the only efficient treatment of the condition is to interrupt gestation. Gestational hypertension diseases prove to be complex; the etiology of PE and HELLP syndrome are not completely understood and preventive treatment remains unknown. A previous HELLP pregnancy is associated with a high risk of developing HELLP (14–24%) and PE (22–28%) in subsequent pregnancies, indicating related pathogenetic mechanisms [69–71].

##### Disease screening

Polak and Mendyk developed at GDM screening tool using ANNs to model relationships between demographic factors and the risk of GDM [30]. In comparison to LR, the ANN model correctly predicted 70% of true positive diagnoses to the LR correct prediction of 56% of true positive diagnoses. Moreira et al. propose application of the radial basis function network (RBFNetwork), an ANN technique, to identify possible cases of GDM in pregnant women and in result achieved 79% precision, and an F-measure of 0.79 [23]. Meant to support hospital management, the RBFNetwork is based on ANN in business intelligence. The authors suggest that a decrease in the prevalence of GDM would reflect the joint effort of pregnant women, experts in healthcare, and healthcare management staff.

There have been efforts to diagnose GDM earlier in pregnancy, when current medical protocols using Oral Glucose Tolerance Test are not feasible. Filho et al. propose a hybrid methodology in order to support the early diagnosis of GDM [24]. The method combines aspects of BNs, Multicriteria Analysis and Expert Systems. The information gathered was structured in the knowledge base of a rule-based specialist system, through Expert SINTA software. The prediction system uses disease code and analysis of medical history of pregnant patients from a database of a health insurance company that covers 11 Brazilian states.

Tejera et al. constructed a model for classification of women with normal, hypertensive and preeclamptic pregnancy using maternal heart rate variability indexes and ANNs [25]. The model obtained around 80% sensitivity for PE, with higher percentage for the normal and hypertensive groups. Liu et al. applied a multi- ‘omics’ approach to develop validated PE biomarkers, comparing serum proteomes in PE and control subjects [21]. In order to construct a sensitive and specific biomarker panel, with the least number of protein analytes, the authors used a genetic algorithm (R genalg package). Multiple biomarkers were discovered, reflecting the complex aspects of PE disease.

Moreira et al. propose a model using ANNs and fuzzy logic to predict HELLP syndrome in high-risk pregnancies [26]. The model combines the learning capacity of ANNs with the reasoning ability of fuzzy systems. As this model was designed with mobile cloud computing in mind, this structure avoids diffuse inference, which requires considerable computational effort. The proposed model performs comparably to other ML methods. In comparison, MLP performs better than the proposed neuro-fuzzy method, but the proposed model has better computational performance.

Asymptomatic bacteriuria (ASB) is known to occur in 2–10% of pregnancies and is associated with preterm birth, low birth weight, and perinatal mortality [72]. Burton et al. tested three ML methods, namely RF, neural networks, and Extreme Gradient Boosting, to reduce diagnostic workload without compromising the detection rate of urinary tract infections [31]. This study made considerations for pregnant patients in the model, after finding classification sensitivity to this subpopulation. Pregnant patients, children, and the rest of the patients in the dataset were trained independently. This increased performance for the pregnant subpopulation in the study. However, other research indicates that the risk of pyelonephritis in untreated ASB is low [73], or observe no increased maternal or neonatal adverse effects in women with untreated ASB [74]. These findings question the use of such screening practices during pregnancy.

##### Disease management with clinical decision support systems

Because patients diagnosed with GDM require more monitoring, it lends itself easily to be supplemented with CDSS to manage the disease. Applications focused on improving home monitoring, therapy planning, dietary recommendations, and patient engagement. Hernando et al. developed a CDSS for the analysis of home monitoring and therapy planning in gestational diabetes [33]. In order to manage the uncertainty in the data, the DIABNET system integrates qualitative and quantitative reasoning. The system was designed for use in patient encounters, supporting the physician by proposing qualitative diet modifications and quantitative changes in insulin therapy. An evaluation of DIABNET found that the system detects the need for a therapy modification in 92% of cases, and expert evaluators accepted 74–86% of the proposals as valid [34].

Caballero-Ruiz et al. developed a web-based telemedicine platform, Sinedie, to remotely evaluate GDM, allowing patients to upload their glycaemia data directly from their glucose monitor and report other monitoring variables [35]. Dietary recommendations are automatically prescribed and notified to patients, while insulin therapy recommendations are notified to the physicians for treatment planning. Glycaema classification is performed based on the EM clustering algorithm and a C4.5 decision tree (DT) algorithm. Sinedie is designed to be sensitive regarding insulin therapy recommendations, in order to avoid false negatives regardless of false positives. In result, the system reduced clinician evaluation time by about 27%, and face-to-face visits per patient were reduced by approximately 89%.

Peleg et al. demonstrate the feasibility of the functionality and architecture of an interactive guideline-based mobile CDSS, MobiGuide, for patient-centered care designed to improve patient engagement [41]. The working prototype was evaluated partially for GDM with and without hypertension in a hospital in Spain. The study demonstrated higher compliance of GDM patients to the computer-interpretable guidelines (CIG), along with an increase in patient and care provider satisfaction. The mobile application for health management could improve financial costs due to a lower rate of complications and hospitalizations, as well as fewer clinic visits for monitoring the disease. Rigla et al. conducted a pilot study to test the feasibility and acceptance of a mobile CDSS, which provides personalized CIG for GDM management [36]. With the mobile CDSS, compliance with blood glucose (BG) monitoring performance was observed higher than with usual care. In result of a questionnaire on the use of the system, the authors found a high level of acceptance.

#### Labor analgesia

Neuraxial labor analgesia is widely used to reduce pain during childbirth, ranging from 37 to 80% in the US [75]. One such neuraxial labor analgesia technique is epidural anesthesia. Yu et al. developed an image classification algorithm that automatically identifies the bone/interspinous region for ultrasound images obtained from the lumbar spine of pregnant patients in the transverse plane [22]. Features were extracted with template matching and midline detection in order to provide a compact description of the ultrasound image. The SVM model was used to classify the interspinous and bone images with maximal margins, with a success rate of 93% on the test set. When further tested on ultrasound video streams, the proposed method correctly identified the site in 45 of the 46 cases. This study focuses on identification of the optimal placement for epidural anesthesia, but no research concerning anesthesia dosing was found.

#### Mode of delivery

Abbas et al. aimed to identify risk factors associated with cesarean sections among women in Muzaffarabad, Jammu and Kashmir. 23 elements with 488 subjects were used for classification, including maternal age, blood pressure, hemoglobin, mode of last delivery, miscarriages, abortions, hypertension, folic acid, diabetes, medicine, heavy breathing, headache, body pain, etc. After applying tenfold cross-validation upon RF, linear discriminant analysis (LDA), SVM, NB, and *k* nearest neighbor (*k*-NN) classifiers, RF proved to perform best for the purpose of this study with highest precision, accuracy, and recall. The analysis revealed that maternal age and the mode of the last birth have a significant effect on the mode of the expected birth. This study included medicine as a feature, considering risk factors for cesarean sections. The specific medicine was not found to be a significant feature in their model.

##### Predict postpartum disease

Maternal outcomes of GDM include the following postpartum: a seven-fold increased risk of developing T2D [76], and increased risk of metabolic syndrome and CVD [77]. A number of studies have applied ML to improve diabetes prediction postpartum in women diagnosed with GDM, proposing feature selection methods and novel machine learning algorithms. Lin et al. applied a supervised learning algorithm to determine whether a pregnant woman has or is likely to develop DM [32]. The aim of the study was to evaluate the feasibility in using the Artificial Immune Recognition System (AIRS) to predict DM development following GDM. AIRS is inspired by natural and artificial immune system mechanisms, including resources competition, clone selection, maturation, mutation, and memory cells generation [78]. Training and test data are seen as antigens in AIRS, and then they induce the B-cells in the system to produce artificial recognition balls (ARBs). The ARBs then compete with each other for the given resource number. ARBs with higher resources will get more chances to produce the mutated offspring to improve the system. After all training antigens have been introduced; the memory cells are generated to classify the test data. Wang et al. proposed a novel method for feature selection by combining EM algorithm with the nearest neighbor classifier [27]. The authors confirmed that the proposed method could effectively predict T2D after a GDM pregnancy in Taiwanese women. Meenakshi and Maragatham evaluated a ML algorithm, the Convolutional Neural Network (CNN), to predict if women with GDM is likely to develop T2D later in life [28]. Despite the reported high performance with 1000 neurons, the CNN model provides no interpretable evidence to predict T2D.

#### Pharmacologics and pregnancy

Efforts have been made to provide patient counselling support and drug information by the support of expert systems. In 1994, Swart, Vos and Tromp proposed a prototype knowledge system to support patients in their encounter with a professional in community pharmacies [39]. In this system, items of information are ranked in order of importance (important, possibly important, and remaining items). In recent efforts, researchers have proposed other methods in order to overcome the cumbersome development and maintenance of rule-based systems.

Boland et al. developed a method that utilizes machine learning to predict the *fetal* toxicity of pharmacologics taken during pregnancy, including first through third trimesters of the pregnancy [79]. The ML method employed a technique called ‘random forests’ whereby information was learned from drugs that were known to be harmful to the fetus by previous outcomes studies (and were already labeled as contraindicated in pregnancy by the United States Food and Drug Administration (FDA)—or category D or X). The model also used information on drugs that were known to be safe to the fetus via previous outcome studies (and labeled as category A or B by the FDA). The AI method was able to predict which drugs were more likely to be fetal toxic versus fetal safe for those drugs that were in the middle or unknown fetal toxicity category (i.e., FDA Category C drugs). Boland et al.’s method also used chemical information on the drugs and whether or not the drug was known to target a vitamin gene or a known Mendelian disease gene to improve the performance of the method [79]. The importance of understanding Mendelian disease genes and pharmacologics that target them (even as unintended targets or ‘off-targets’) in *fetal* outcomes was established via an extensive manual review process [80] that helped to inform the design of our later machine learning approach [79].

Souissi et al. propose a recommendation system for antibiotic prescription, PARS [40]. While this recommendation system was not developed specifically for use during pregnancy, a use case of a pregnant woman is demonstrated. The system depends on an antibiotic ontology, an infection ontology, and a patient ontology. This information is then used in two reasoning stages, which then output a final set of recommended antibiotic treatments, personalized for the patient. The CDSS combines these ontologies with MCDA (Multiple Criteria Decision Aiding) for knowledge-driven treatment. An ontology-focused approach was chosen over ML due to the fact that it is not currently able to provide recommendations with complete explanations, which is crucial in medicine. Ontologies and MCDA in this model can provide a full explanation with a treatment recommendation and do not depend on a training set. PARS is limited to bacterial infection prescriptions and not applicable to other cases of antibiotic use. Moreover, while PARS considers toxicity risk of antibiotic treatments, it does not consider drug-drug interactions with ongoing therapies for the patient. The authors plan to integrate other ontologies in order to address these limitations of the CDSS.

### Future applications of AI for maternal and fetal health outcomes

The future of AI in medicine, specifically in women’s health, should focus on informing pregnant women and their physicians on the maternal and *fetal* consequences of pharmacologics taken during pregnancy. This would allow for better and more informed decision making both for the patient and the physician. ML is one AI tool that can be used to enable better science to be generated from our existing datasets. Efforts have been made to improve drug discovery [81, 82], and pharmacovigilance [83], but few address drug safety during pregnancy.

#### Translational human research opportunities

Once we have sound and reliable information on whether a drug is thought to be harmful either to the fetus or the mother, we will be able to design better animal experiments to validate our hypotheses. ML can also be used to aid and assist in designing and optimizing animal experiments that are specific to the clinical question and outcomes anticipated (e.g., pigs are thought to be more similar to humans with regards to their skin— this information could be harnessed by ML to assist in designing animal studies).

#### Optimizing drug dosage

Physiological changes during pregnancy are known to alter overall systemic drug exposure. This is especially important for pregnancy-associated disease and chronic disease management during pregnancy. Our query found one paper on SLE, but this is a single example of chronic health conditions managed during pregnancy. Applications of ML to inform drug dosing during pregnancy did not arise from our query, illustrating the need to address this gap. Efforts have been made concerning other health issues, such as warfarin dosage [84], and radiation oncology [85]. Moreover, there is a significant gap between accumulating knowledge of pregnancy-associated pharmacokinetics changes and our understanding of their clinical impact for the mother and fetus [86].

#### Opportunities in clinical decision support

Once the science has been created (using ML and existing clinical datasets) and validated (using ML and optimizing animal experiments)—the results can then be used to inform pregnant women and their physicians. We can also use AI methods for this last stage in the pipeline (Fig. 2). Effective clinical decision support requires AI methods to optimize the delivery of the information to the physician at a point in the clinical workflow that is optimal for decision-making purposes. If the information is presented too late, then the physician will not be able to inform the patient because the patient would have left already. If the information is presented too early, the patient may not be pregnant yet and therefore the information may not be helpful. Therefore, appropriate timing of information is required to optimize the clinical workflow process. Currently, many alerting systems that use clinical decision support overlook the power of AI and rely heavily on outdated AI ‘expert’ system methods that use hardcoded decision trees. Dynamic AI-powered rule-based decision tree methods can be used that learn from the users to refine and optimize the presentation of the information to the clinician. If the decision-making algorithms are optimized using smarter AI methods, both physicians and their patients will be able to be presented with the right information at the right time.

AI has the power to transform healthcare, specifically women’s health. Hopefully the next 25 years will see strides in terms of incorporating modern AI methods in all aspects of the women’s health space from a.) obtaining sound and reliable data from clinical records, b.) designing optimized animal experiments to validate specific hypotheses to c.) implementing decision support systems that inform physicians and their patients for shared patient decision-making.

### Limitations

Most studies included cross-validation, but external validation was limited. This impedes on generalizability of the studies. Another limitation stems from the fact few applications of AI are focused on pharmacological treatment. Many excluded papers were focused on other applications for maternal reproductive health, such as methods to extract fetal signals from monitoring records (i.e. echocardiogram), or methods for screening for fetal chromosomal and congenital abnormalities. This demonstrates the great amount of effort to improve fetal monitoring and fetal screening. However, it highlights the need to apply AI and machine learning methods to other aspects of the maternal reproductive healthcare spectrum. We focused our review on pregnancy as a recent review on lactation was published [87]. Therefore, our review does not assess the role of AI methods in lactation studies. A future review could explore the potential for AI on pharmacologics effects on lactation as this is an important area.

## Conclusion

Our review demonstrates how AI has been applied to address pharmacological exposures during pregnancy and this includes the entire pregnancy process: preconception, prenatal, perinatal, and postnatal health concerns. We identify three areas where AI methods could be used to improve our understanding of pharmacological effects of pregnancy, including: a.) obtaining sound and reliable data from clinical records (15 studies), b.) designing optimized animal experiments to validate specific hypotheses (1 study) to c.) implementing decision support systems that inform decision-making (11 studies). The largest literature gap that we identified is with regards to using AI methods to optimize translational studies between animals and humans for pregnancy-related drug exposures. However, in general all 3 areas were lacking research regarding the pharmacological exposure-pregnancy aspect with less than 20 studies per category. Incorporating modern AI methods into understanding the maternal and fetal consequences of pharmacological drug exposure is a must for future studies. Applications of AI to other aspects of pregnancy, maternal, and fetal health, including lactation can inform the necessary research to delve more deeply into how pharmacologics affect pregnancy.

## Electronic supplementary material

Below is the link to the electronic supplementary material.

Supplementary file1 (PDF 89 kb)

## Abbreviations

AI: Artificial intelligence
ML: Machine learning
ANN: Artificial neural network
BN: Bayesian Network
CDSS: Clinical decision support system
DL: Deep learning
DT: Decision tree
EM: Expectation–maximization
k-NN: K nearest neighbor
LDA: Linear discriminant analysis
LR: Logistic regression
MLP: Multi-layer perceptron
NB: Naïve Bayes
RF: Random forest
SVM: Support vector machines
