# Supplementary material for: Enabling pregnant women and their physicians to make informed medication decisions using artificial intelligence
Source: J Pharmacokinet Pharmacodyn. 2020 Apr 11;47(4):305–18. doi: 10.1007/s10928-020-09685-1 (PMC7473961; doi:10.1007/s10928-020-09685-1)
Supplement: Supplementary file 1 — Supplementary file1 (PDF 89 kb) [file 10928_2020_9685_MOESM1_ESM.pdf]

Supplemental Tables for:

Davidson LM, Boland MR.

Enabling Pregnant Women and their Physicians to Make Informed Medication Decisions Using Artificial Intelligence

Table of Contents

Tables

Table S1. Summary of Journals with Studies Included in Our Review..………………...………..……................2

Table S1. Summary of Journals with Studies Included in Our Review

General Topic	Journal	Number of papers
Informatics	International Journal of Medical Informatics	3
	BMC Medical Informatics and Decision Making	1
	Journal of Biomedical Informatics	2
	Medical Informatics	1
	Artificial Intelligence in Medicine	1
Computational and Engineering	Expert Systems with Applications	1
	Medical & Biological Engineering & Computing	1
	Neural Computing and Applications	1
	Perspectives of Pattern Recognition	1
	Computer Methods and Programs in Biomedicine	1
	Journal of Computational Science	1
	Concurrency Computation	1
Reproductive Medicine	Systems Biology in Reproductive Medicine	1
	Human Reproduction	2
	The Journal of Maternal-Fetal & Neonatal Medicine	1
Birth Defects	Teratology	1
Imaging	Ultrasound in Medicine and Biology	1
Pharmacy Science	Pharmacy World & Science	1
	Research Journal of Pharmacy and Technology	1
Health Information	npj Digital Medicine	1
	Journal of Medical Systems	1
Medicine & Biology	Journal of Diabetes Science and Technology	1
	In vivo	1
	Current Hypertension Reports	1
	BMC Medicine	1
	Molecular and Cellular Proteomics	1
General Science	IEEE Access	1
